# The technology, opportunities, and challenges of Synthetic Biological Intelligence

**DOI:** 10.1016/j.biotechadv.2023.108233

**Published:** 2023-08-07

**Authors:** Brett J. Kagan, Christopher Gyngell, Tamra Lysaght, Victor M. Cole, Tsutomu Sawai, Julian Savulescu

**Affiliations:** 1 Cortical Labs, Melbourne, VIC, Australia; 2 Murdoch Children’s Research Institute, Melbourne, VIC, Australia; 3 University of Melbourne, Melbourne, VIC, Australia; 4 Centre for Biomedical Ethics, Yong Loo Lin School of Medicine, National University of Singapore, Singapore; 5 Graduate School of Humanities and Social Sciences, Hiroshima University, Hiroshima, Japan,; 6 Institute for the Advanced Study of Human Biology (ASHBi), Kyoto University, Kyoto, Japan; 7 Oxford Uehiro Centre for Practical Ethics, Faculty of Philosophy, University of Oxford, Oxford, United Kingdom

**Keywords:** Biocomputing, neuroscience, synthetic biology, intelligence, ethics

## Abstract

Integrating neural cultures developed through synthetic biology methods with digital computing has enabled the early development of Synthetic Biological Intelligence (SBI). Recently, key studies have emphasized the advantages of biological neural systems in some information processing tasks. However, neither the technology behind this early development, nor the potential ethical opportunities or challenges, have been explored in detail yet. Here, we review the key aspects that facilitate the development of SBI and explore potential applications. Considering these foreseeable use cases, various ethical implications are proposed. Ultimately, this work aims to provide a robust framework to structure ethical considerations to ensure that SBI technology can be both researched and applied responsibly.

## Introduction

Advancements in hardware, software, and synthetic biology (wetware) have resulted in new methods for interacting with *in vitro* biological neural systems. The most advanced of these have sought to embody these neural systems into simulated environments to elicit dynamic goal-directed behavior, referred to as Synthetic Biological Intelligence (SBI)(Kagan et al., 2022b). SBI systems can be broadly defined as the result of intentionally synthesizing a combination of biological and silicon substrates *in vitro* for the purpose of goal-directed or otherwise intelligent behavior^1^. SBI is distinct from brain-computer interface (BCI) and similar approaches as it does not involve whole organisms, using only specific biological material, usually neural tissue derived typically through synthetic biology processes, as a biomimetic material within the larger system.

It is only relatively recent that the ethics of experimenting with brain tissue has been seriously considered, with the overwhelming focus on cells from a human origin(Farahany et al., 2018). The majority of these ethical considerations also focus on the generation of 3-dimensional (3D) neural structures generally referred to as “organoids” derived from human stem cells(Hostiuc et al., 2019; Koplin and Savulescu, 2019; Lavazza, 2021; Lavazza and Massimini, 2018; Sawai et al., 2021, 2019). Typically, these discussions do not account for the significant variability amongst different organoids or that a continuum exists between simpler monolayers of neural tissue and various assemblies of more complicated organoids. The argument for more focused consideration on the organoid structure is usually based on the assumption that greater complexity alone may lead to qualitatively different traits when compared to monolayers (Hostiuc et al., 2019; Koplin and Savulescu, 2019; Lavazza, 2021; Lavazza and Massimini, 2018; Lavazza and Reichlin, 2023; Sawai et al., 2021, 2019). If so, it is also crucial that future work considers where this complexity reaches a level requiring such consideration. Contrary to the binary use of language where cell cultures are described either as ‘monolayers’ or ‘organoids’, the structures can vary massively in terms of size, cellular diversity, and complexity – requiring far more nuanced considerations than what is typically adopted (Friston, 2023; Gabriel et al., 2021; Miller, 2023; Miura et al., 2020). This discourse is further complicated by inconsistencies in terminology and nomenclature even when referring to similar cell structures, and uncertainties around the ontological and potential moral status of these structures(Hostiuc et al., 2019; Kagan et al., 2022a, 2022a; Koplin and Gyngell, 2020; Koplin and Savulescu, 2019; Sawai et al., 2021). Here, we outline details of SBI as an emerging technology, along with the foreseeable applications and ethical considerations that may arise. Finally, we propose a pathway for promoting constructive dialogue and adopting an ethical approach that balances potential utility with foreseeable risks of harm and the uncertainty inherent to novel technologies.

## The Development of Closed-Loop Systems to Embody *in vitro* Neural Systems

The use of closed-loop paradigms for *in vitro* neurons – whereby activity from a neural system is measured, applied to an environment, and updated environmental information communicated back to the neural system – has received relatively limited exploration. Early work supported the proposition that *in vitro* neurons would respond to incoming stimulation adaptively or engage in behaviors consistent with blind-source separation phenomena (Isomura et al., 2015; Shahaf and Marom, 2001). Following on from this, several studies developed tools for, or identified interesting neural response patterns from, *in vitro* closed loop stimulation paradigms, e.g.(Brewer et al., 2013; Müller et al., 2013; Newman et al., 2013; Pimashkin et al., 2013; Rolston, 2009; Tessadori et al., 2012). Preliminary investigations into goal-directed *in vitro* neural behavior displayed limited robustness or details which precluded any conclusion of goal-directed learning and/or did not pass through full independent peer review (e.g. (Aaser et al., 2017; George et al., 2015; Masumori et al., 2020; Reger et al., 2000)). Yet key work demonstrated that closed-loop stimulation resulted in significantly greater functional plasticity over time and potentially exhibited some other shaped behavior (Bakkum et al., 2008a, 2008b; George et al., 2018; Tessadori et al., 2012).

Building on this work, recent research has shown that *in vitro* biological networks of cortical cells, from either mouse or human origin via synthetic biology methods, were able to display real-time adaptive goal-directed learning in simulated environments(Kagan et al., 2022b). Importantly, this work outlines key methods and hypotheses which can identify the potential mechanism of actions behind goal-directed or intelligent behaviors in neural systems. Interestingly, the results accorded with multiple electrophysiological changes that were also observed. Intelligence, displayed through the goal-directed behavior of embodied^2^ *in vitro* neurons, was termed SBI. As an umbrella term, SBI has unique properties that open key considerations previously less critical to consider. Three key factors can be identified as technological preconditions of SBI: 1) the scalable and diverse opportunities that arise from modern stem cell technology and synthetic biological methods; 2) the hardware and software applications which enable the interaction with the biological tissue; 3) the neurocomputational theories and subsequent inferences for eliciting behavior from the system and to better understand what the implications of this may be.

### 1) Stem Cell Technology & Synthetic Biology

Perhaps the largest advancement in experimental neurobiology related to SBI has occurred with the generation of renewable pluripotent stem cell cultures that can be differentiated to neural cells(Hu et al., 2010). Early work was performed via embryonic stem cells(Dottori and Pera, 2008; Liu et al., 2018; Shi et al., 2012), yet the later generation of induced pluripotent stem cell (iPSC) lines, generated from consenting donations of adult tissue, provides an ethical and renewable process for generating neural tissue(Chambers et al., 2009; Kim et al., 2021; Pauly et al., 2018; Pawlowski et al., 2017; Shi et al., 2012). Most previous work interacting with neural tissue focused on primary cell culture, whereby neurons were obtained from living animals, disassociated, and grown under controlled conditions. While this does produce viable neural cultures and can be somewhat specific depending on the technical quality of those performing the work(Kaech and Banker, 2006; Lossi and Merighi, 2018; vanPelt et al., 2004; Wagenaar et al., 2006, 2004), it has distinct limitations.

Firstly, primary cell culture is, at best, linearly scalable, which means to scale up systems would require a growing number of animals to be killed for tissue harvesting – an ethically fraught prospect (Lossi and Merighi, 2018). Secondly, there are limitations in accessing pure or specific populations of cell types. While broad regions, such as hippocampus or cortex, can be targeted, the ratio of cell types and almost any other factor are difficult to modify. Further, although some organotypic cultures can be generated from primary tissue, the scaling and complexity of these remain limited(Gogolla et al., 2006; Schukking et al., 2018). Finally, the need to breed, house, maintain and harvest neuronal tissue from animals creates a number of logistical, ethical and practical challenges. Deriving neuronal tissues from animals is thus not suited for widespread application and testing of SBI.

**Fig. 1:**
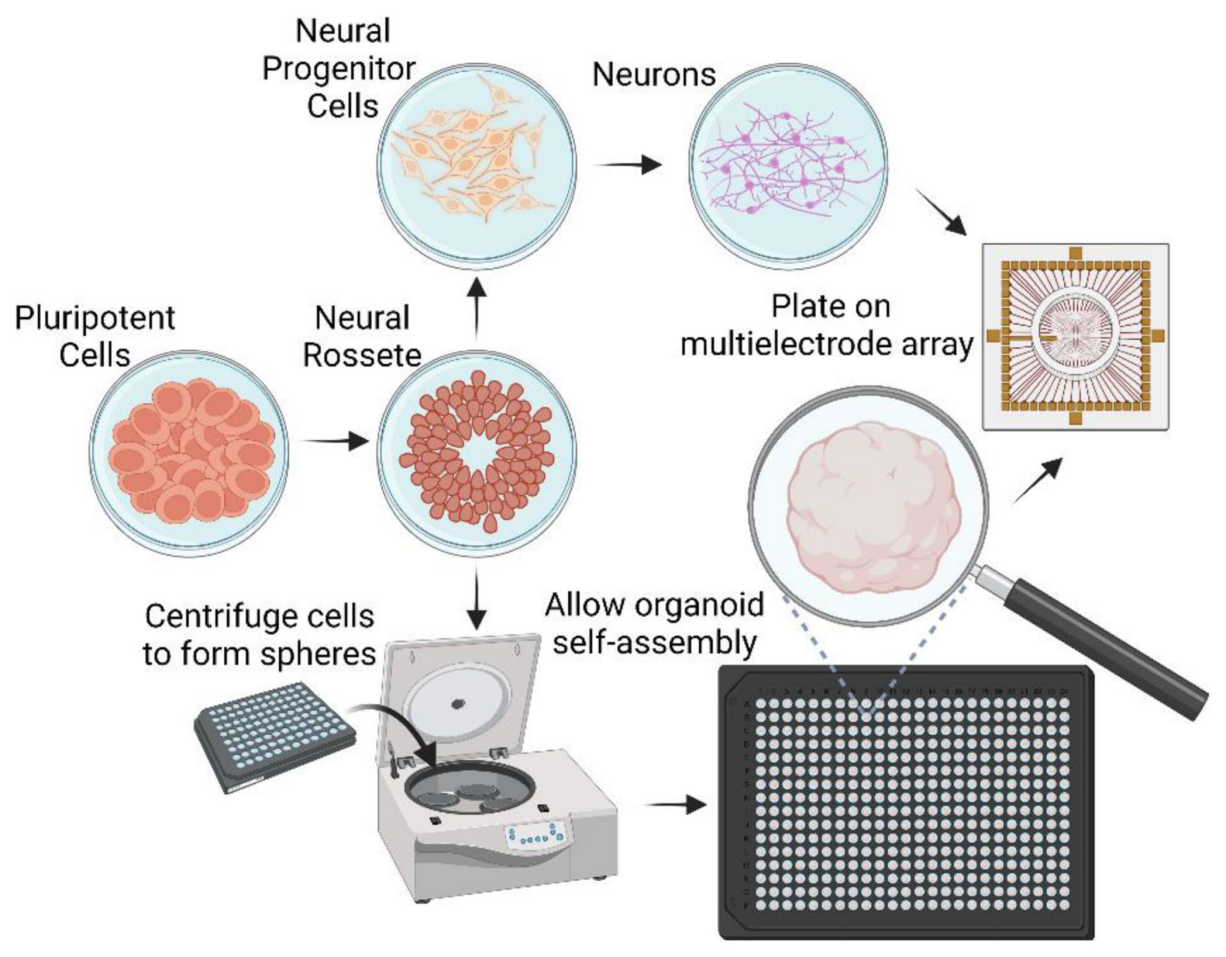
A schematic of key steps and differences between generating a culture of neurons from pluripotent stem cells to 2D (monolayers) compared to 3D (organoids). The essential difference is to allow organoid self-assembly in low-adherence plates after mild centrifugation of cells at early stage of differentiation.

In contrast, the use of iPSCs removes all these concerns while providing new opportunities. Techniques to exponentially scale up the production of iPSCs are well established(Schwedhelm et al., 2019). Neural cells can be generated from iPSCs using methods that follow natural ontogeny (i.e. (Chambers et al., 2009; Shi et al., 2012)), with direct differentiation techniques using viral vectors to modify gene expression (i.e. (Ho et al., 2016; Pak et al., 2018)), or through direct genetic modification to make cell lines overexpress these genes in response to small molecules (Pawlowski et al., 2017). Furthermore, increasingly complex 3D structures (organoids, see Figure 1) can be reliably generated from iPSCs that open up yet further opportunities and challenges(Kim et al., 2020; Miura et al., 2020; Oliveira et al., 2019; Pollen et al., 2019; Sun et al., 2021; Yakoub and Sadek, 2018). Finally, although technical expertise and equipment is still required to generate these neural cultures, the logistical and space requirements are significantly less than involving animal subjects. These advantages of using iPSC tissue for SBI are critical in providing a viable pathway towards wider research and development of the technology above what has previously been done.

### 2) Enhanced Hardware & Software applications

SBI technology must be able to record activity from living biological neurons, transmit this information to a virtual or physical system to allow action, and then provide information back to the biological neural network that can be altered according to the action performed. Ideally this closed loop occurs in real-time, so that the neural system is able to dynamically adapt to the effect of its actions on the environment. Improvements in hardware and software allow for more advanced and nuanced interactions with neural systems.

The most prevalent method of interaction remains through electrophysiological recording and stimulation via multielectrode arrays (MEA)(Jimbo et al., 2003, 1998; Shahaf and Marom, 2001; Wagenaar et al., 2004). However, optic approaches have also been explored(Sun et al., 2019). Previously, limitations in computational power or algorithm efficiency required work to either make sacrifices as to what could be implemented computationally in these systems (i.e., (Tessadori et al., 2012)) or were unable to implement real-time closed-loop systems, requiring relatively long latencies (i.e., (Bakkum et al., 2008b)). Advancements in computational processing power allow greater degrees of data management for signals both in and out of the neural system(Anden and Mallat, 2014; Gacic et al., 2004; Insel et al., 2013; Markiewicz et al., 2021; Mboup, 2012; Smirnova et al., 2023).

Further, while passive MEA are capable of being used in sophisticated approaches(Jimbo et al., 2003; Newman et al., 2013; Rolston, 2009), the development of high-density MEA (HD-MEA) utilizing CMOS technology enabled magnitudes more spatial resolution and flexibility(Heer et al., 2007, 2005, 2004; Jenkner et al., 2004). Future work now focuses on expanding from two-dimensional arrays to better record from and stimulate 3D structures such as organoids (Kalmykov et al., 2019; Park et al., 2021). These advances can be combined with better big data processing pipelines and tools to better analyze and interpret neural activity, including applying machine learning approaches in novel ways (Carlson and Carin, 2019; Insel et al., 2013; Unakafova and Gail, 2019; Yatsenko et al., 2021). The combination of these approaches provides a far greater ability to interact with biological neural networks and then analyze the subsequent outcomes to enable greater expressions of SBI.

### 3) Neurocomputational Theories and Informatic Analysis

While the ability to generate neural tissue and interact with it via hardware and software is necessary for SBI, it is not sufficient. It is also critical to be able to understand mechanisms by which neural systems engage in intelligent and/or goal-directed behavior in order to elicit these functions in a meaningful way. Other works cover the myriad of theories postulated in greater detail (e.g. (Ebitz and Hayden, 2021; Friston, 2010)), so here we provide only a brief overview.

**Fig. 2:**
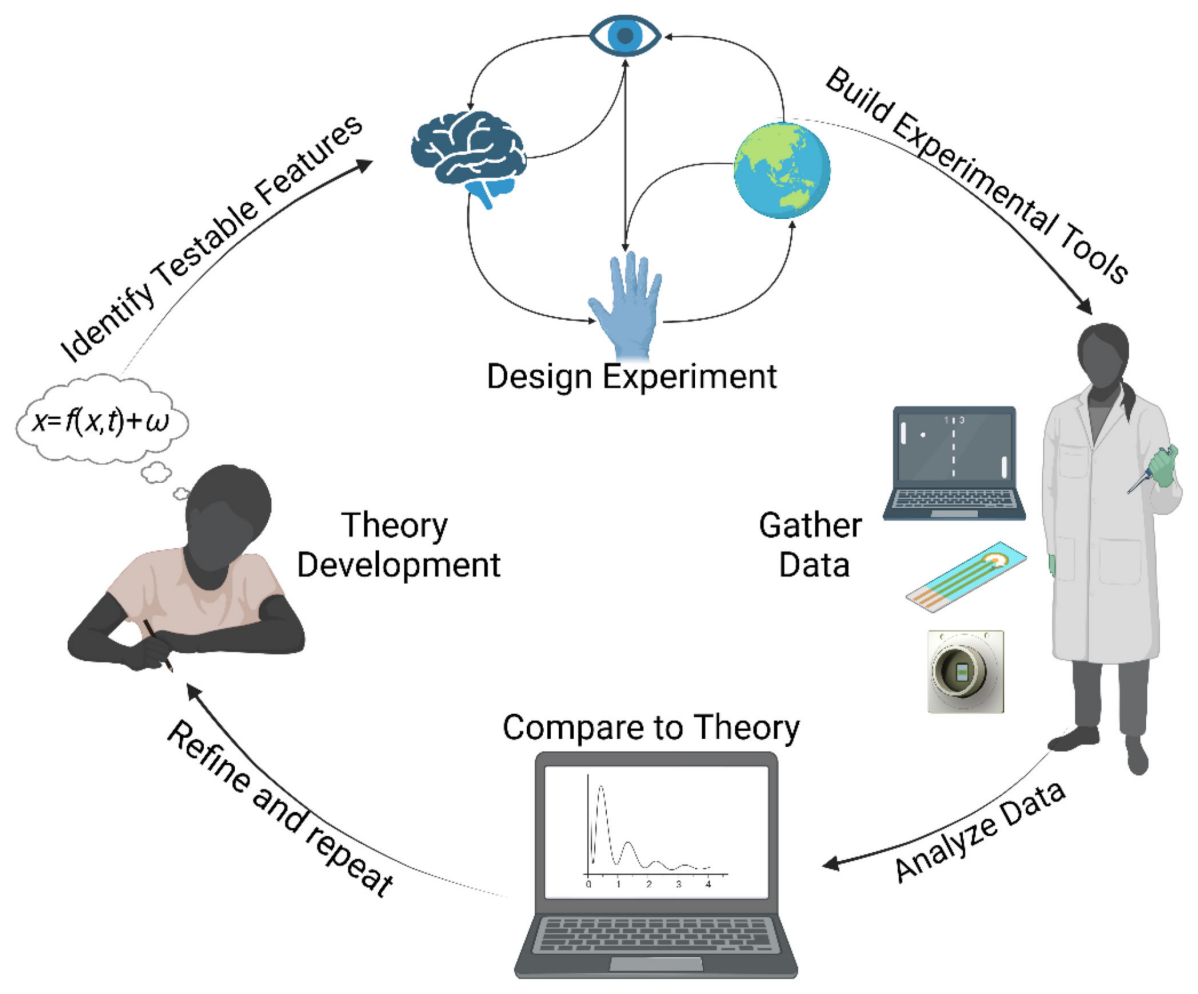
Representation of co-development of theory and experimental tools which can be informed via theory development, identifying testable implications, and designing experiments to test these implications. Experimental tools can then be built and used to generate data which can be compared to the theory and the theory then refined so the process can be repeated.

Theories can either focus on organization or optimization, with the opportunity for overlap. The former attempts to explain the structural and/or functional patterns observed in neural systems (e.g. (Beggs and Plenz, 2003; Clawson et al., 2017; Ebitz et al., 2020; Ly et al., 2012; McDonnell and Stocks, 2008; Poirazi and Papoutsi, 2020; Shew et al., 2015; Toyoizumi et al., 2014)). The latter focuses on why a neural system may exhibit such organization – i.e., why such features are optimal for a system to survive and thrive in a dynamic environment (e.g. (Barlow, 2012; Barto et al., 2013; Friston et al., 2012, 2018, 2009; Kangassalo et al., 2020; Madhav and Cowan, 2020; Schwartenbeck et al., 2019, 2015; Schwartz, 2016; Sinapayen et al., 2017)). One of the limitations of this area is that many theories about how internal states such as intelligence, cognition, sentience, consciousness et cetera may arise and the implications of this are exceedingly difficult to empirically test and interrogate *in vivo*(Friston, 2023; Goddard et al., 2023; Lobov et al., 2023; Pereira et al., 2023). Therefore, while enormous conceptual advancements have been made in this area that can potentially facilitate basic SBI, the ability to test these theories requires SBI techniques to co-develop more controlled research methods (Figure 2). In turn, this will also lead to more advanced applications of SBI.

## Establishing Synthetic Biological Intelligence as an Ethical Platform Technology

While development on each of these areas has been ongoing, the innovation through synthesis enabled by combining these technologies has exceptional promise on multiple fronts. Perhaps for this reason, numerous large national and international research consortia have recently arisen to investigate this area, including: *The Mind in Vitro* project, for which The National Science Foundation awarded a 7-year, $15 million project grant to the multi-university team led by the University of Illinois Urbana-Champaign (UIUC); the EU-funded NEU-ChiP project which received €3.5 million in funding from the European Commission; and the John Hopkins University-led Organoid Intelligence research focus group(“An NSF Expedition in Computing Mind in Vitro Computing with Living Neurons | Mind in Vitro | UIUC,” 2022; “Neuronal networks from Cortical human iPSCs for Machine Learning Processing- NEU-ChiP | NEU-ChiP Project | Fact Sheet | H2020 | CORDIS | European Commission,” 2020; Hartung et al., 2023; Morales Pantoja et al., 2023; Smirnova et al., 2023). Industry-backed research interests have also arisen and are actively involved in pursuing this research, such as Australian based Cortical Labs and USA based Koniku(Hernandez, 2022; Kagan et al., 2022b; “Nerve cells in a dish can learn to play Pong,” 2022).

Preliminary studies have already attempted to integrate these neural systems into both real-world applications through robotics and into virtual environments (e.g. (Bakkum et al., 2008b; Kagan et al., 2022b; Tessadori et al., 2012)), although more work is required. Improvements in SBI technology could allow more useful interactions and processes in these environments. While it is difficult to set likely timelines on when this technology will mature, there are compelling reasons to foresee SBI as a cornerstone of real-time autonomous systems. Biological systems display tremendous capacity to navigate complex and dynamic environments with significant flexible storage, engage in highly sample efficient learning, recover functionality despite significant injury or disease to the brain, and achieve this with minimal power consumption(Herculano-Houzel, 2012; Orger and de Polavieja, 2017; Strubell et al., 2019; Wu et al., 2020). Even current SBI, while rudimentary, has already demonstrated higher sample efficiency compared to deep reinforcement learning algorithms(Habibollahi et al., 2022a). With future work planned to focus on moving towards utilizing organoids as a substrate for intelligent processes, this potentially raises the capabilities even further(Smirnova et al., 2023). As such, the potential of SBI systems has already been recognised as a promising pathway to intelligent systems, especially when real-time, sample efficient, adaptive learning is required (Buchanan, 2018; Grozinger et al., 2019).

Some ethical and regulatory issues raised by the development of SBIs will mirror those raised by brain organoids. This includes concerns around obtaining informed consent from donors(Boers and Bredenoord, 2018; Greely, 2021), ensuring that the privacy and anonymity of donors is protected, developing fair arrangements in relation to the ownership and commercialization of results(Boers et al., 2016; Bredenoord et al., 2017), and managing long-term storage of samples (Farahany et al., 2018; Hyun et al., 2020). Given the potentially complex functionalities of SBI systems, informed consent will be particularly challenging. Ensuring donors are well-informed of the specific implications of SBI’s, having the opportunity to reflect on the risks and benefits, and being able to negotiate fair composition for their donation, will help address these concerns.

However, these ethical restrictions should not be a barrier to research with SBIs, especially in the early stages. The non-invasive nature of donating skin/saliva cells or the minimal risk of donating small amounts of blood, along with the potential to use a small number of donors to generate a large number of SBIs, should mean there is an adequate supply of samples. Yet other issues, such as the legal status of these devices, that are also currently under discussion for brain organoid research will still need to be considered (Jowitt, 2023; Kataoka et al., 2023)

Rather than looking deeper at the donor issues raised by SBIs, we will focus on other issues which have received less attention. These can be broadly broken down into two key subsets: 1) concerns about applications of SBI technology; 2) uncertainty around the potential of SBI technology to give rise to ‘conscious’ systems that may be worthy of special moral consideration. We discuss both below.

## Ethical Considerations using SBI for Disease Modeling and Drug Testing

A key short-term benefit could focus on potentially more advanced *in vitro* preclinical drug screening and modeling of brain-related diseases or disorders. Recently, *in vitro* testing drug targets has become increasingly more common, especially with the advent of organoids(Benam et al., 2016; Fusco et al., 2019; Neužil et al., 2012, p.; Thodeson et al., 2018). Yet while this work can be very effective in some instances, ultimately for diseases where neurological and psychiatric factors are involved, they do not capture the essential function of a neural system. Simply put, the purpose of a neural system is not to express key markers of display firing, it is to process information and respond accordingly, typically in a dynamic fashion.

For this reason, historically this work has been conducted on animals, specifically rodent models e.g., (Golyala and Kwan, 2017; Lossi and Merighi, 2018; Ma et al., 2019; Ocampo et al., 2016; Putnam and Merritt, 1937). Rodent models have some physiological similarities to humans, yet are extremely low throughput solutions and require expensive support personnel and infrastructure to maintain(Henry and Wlodkowic, 2019; Tomasello and Wlodkowic, 2022). Conversely, other models, such as zebrafish are much higher throughput, yet have fewer physiological linkages to humans(Henry and Wlodkowic, 2019; Tomasello and Wlodkowic, 2022). Brain organoid models have already been used as an alternative to animals in research on neurological diseases(Qian et al., 2017). Integrating lab-grown neurons into SBIs may enable a wider range of medical research to occur within *in vitro* models. SBI offers the potential to create high-throughput models of brain disease that are physiologically similar to humans, facilitating better research into brain disease and pre-clinical drug screening, and doing so while reducing the need for animal suffering(Fusco et al., 2019; Habibollahi et al., *in press;* Haring et al., 2017; Myers, 2017; Tejavibulya and Sia, 2016). Despite this promise, the translatability of this approach will still need to be carefully assessed to ensure safety and external predictive validity(Hyun et al., 2020).

One challenge with using stem cell models for drug screening is a lack of diversity in stem cell lines(Ghosh et al., 2022). Current stem cells lines are predominately made from cells of people with European ancestry. As drug responses can differ amongst people of different genetic backgrounds, results from stem cell models created from a single cell line may not be generalizable. This limitation raises concerns in relation to equity and justice. A short-term solution to this problem is to ensure SBIs are created using multiple stem cell lines from people with diverse genetic ancestries. A medium-term solution is to combine SBIs with personalized medicine approaches to the study and treatment of brain disease, by allowing SBIs to be grown from patients’ own cells which then exactly match their genotype. As drug responses can differ from individual to individual, the personalized medicine approach is particularly promising (Miller et al., 2001).

In this manner, SBI offers benefits both in potentially providing advanced pathways in disease modeling and testing novel therapies with the chance to see how metrics related to information processing are impacted. While an equity issue may still exist around access to this personalized approach, here the early involvement of industry research is a potential advantage. Industry inherently has a predisposition to work towards more affordable solutions to enable access to broader markets. Therefore, although industry research partners into SBIs may be incentivized to reduce access barriers through self-interest and reduce concerns around equity, further exploration of this issue is required.

Coupled with the above, a related ethical benefit is that SBIs may reduce the need for animal testing in certain cases. Given the animals whose cognition most closely resembles human cognition (non-human primates(Aguilera et al., 2021; DeGrazia, 2016; Phillips et al., 2014)) are also the animals whose use in testing raises the greatest moral concern (e.g. see (Carvalho et al., 2018)), this application of SBIs can be viewed as strongly ethically desirable (Gyngell et al., 2022; Kagan et al., 2022a). A general principle of research ethics is that we should aim to minimize risk of harm to research participants(“World Medical Association Declaration of Helsinki,” 2013). One of the ways in which this principle can be operationalized is by ensuring testing occurs in entities that have the lowest moral status. This is sometimes called the ‘subsidiarity principle’ and has previously been used to argue that we only should avoid testing on embryonic stem cells where the same tests can be performed using other stem cells with fewer moral concerns to consider (Pennings, 2004). This same principle can be used to argue that we should be testing on SBIs rather than animals wherever possible and would emphasize the ethical merit of this endeavor.

## Ethical Considerations using SBI for Computational or Intelligent Processes

Developing SBI also offers the potential to better understand how computation or intelligence arises in neural systems. This exploration offers both short- and long-term applications. Shorter term SBIs offer the chance to explore how neural systems process information and provide the potential to refine existing, or develop new, theories. Being able to better understand how neural systems display traits such as ‘intelligence’ also means that such traits could be leveraged in wider applications in the future. Such applications could focus on improving drug discovery, better understandings of neural function, or even advanced iterations of SBI technology, perhaps being leveraged for real-time autonomous tasks in robotics.

**Fig. 3:**
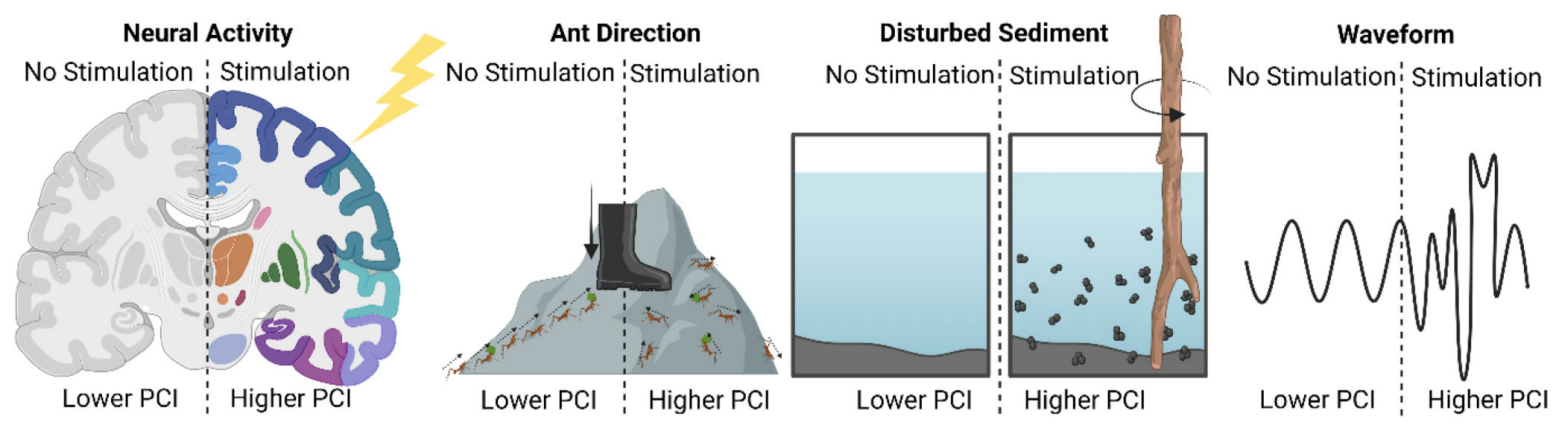
Simplified comparison for how the Perturbational Complexity Index (PCI) as a metric is not inherently a suitable marker for consciousness, whereby a PCI metric would be increased after stimulation of several systems, yet not all systems could reasonably be considered conscious.

As part of this work, from an ethical perspective, it is also necessary to consider neurocomputational and informatic approaches that try to quantify when a neural system may also display a trait requiring moral attention. Approaches such as the Integrated Information Theory (IIT), neuro-representationalism, active inference, global workspace theories (GWTs), et cetera., offer avenues to establish useful correlates of potential states (Parr and Friston, 2017; Seth and Bayne, 2022; Tononi et al., 2016; Tononi and Koch, 2015). Moreover, compelling neural correlates of consciousness in humans have been previously proposed, such as the Perturbational Complexity Index (PCI) or neural criticality, which offer other approaches to consider(Habibollahi et al., *in press;* Kagan et al., 2022a; Sinitsyn et al., 2020; Toker et al., 2022). Yet assumptions behind these approaches means these metrics can have serious limitations in predictive validity if inappropriately applied to *in vitro* (or other) systems as similar mathematical criteria could be established in non-conscious systems (see Figure 3 for examples)(Habibollahi et al., *in press;* Kagan et al., 2022a; Sinitsyn et al., 2020).

Further applications of SBI research are aimed at developing approaches to integrate broader theories of population dynamics with more reductive single cell processes, to allow a better understanding of neurobiology(Ebitz and Hayden, 2021; Mattar and Lengyel, 2022). For example, early work has already identified conditions which give rise to traits such as neural criticality *in vitro*, previously a contentious question(Habibollahi et al., *in press*). Not only would this inform fundamental mechanisms underpinning intelligence and related states, it may provide insight into more efficient or powerful algorithms for machine learning and artificial intelligence research – consistent with calls from the research community(Zador et al., 2022).

A potential longer-term benefit of SBI research is more sustainable computer systems which are less dependent on the availability of large amounts of power to operate. Climate change, driven by increasing carbon emissions, has been described as the greatest moral challenge of our time(MacDonald and Sloman, 2020). It results in direct harm to individuals through extreme weather events and supply disruptions for essential resources. Furthermore, the burden of climate change falls predominantly on those living in low-income countries and raises serious concerns about global justice. Biological intelligences are much more energy efficient than traditional computer systems, with a human brain approximately using 20 watts of energy, able to be distributed through a complex network (Balasubramanian, 2021; Palombit et al., 2022; Raichle and Mintun, 2006; Ramchandran et al., 2019). In contrast, consider the K supercomputer produced by Fujitsu, which can perform 8.2 billion megaflops (1,048,576 floating-point operations per second) but which requires 9.9 million watts to be powered. The increased use of computer systems in all aspects of our lives has led to increased carbon emissions coming from the IT industry(Freitag et al., 2021). These problems will be exacerbated by the increased use of machine learning algorithms and systems of generative artificial intelligence, which often require power intensive super-computers to operate (Jouppi et al., 2020). As such, if even a small proportion of these information processing tasks can be done with SBI, there is a compelling environmental reason to explore these alternatives.

## How to Approach Additional Ethical Considerations for SBI

Foremost, it is imperative that a broadly agreed upon nomenclature for this field is adopted(Kagan et al., 2022b; Pereira et al., 2023; Rommelfanger et al., 2023). We used the words conscious and intelligence above in quotation marks precisely because there are different ways of understanding these terms with different implications for how we describe SBIs(Graziano, 2021; Pereira et al., 2023). It is preferrable that the field has agreed terms to describe the different aspects of SBI to enable constructive discussions and exploration of the technology, along with considering the ethical challenges. Without at least broad standardization^3^ of terms, constructive discourse will be greatly hampered. Previously, key terminology has been imprecise, with signifiers used interchangeably to represent one or another concept that are themselves seldom formally defined. Even in cases where a term may be defined in one paper, the lack of coherence in the field can give rise to semantic disagreements that may distract from underlying scientific efforts(Kagan et al., 2023; Rommelfanger et al., 2023; Wallis, 2022). Terms related to complex processes or internal states that are attributed various degrees of moral status are particularly challenging. These include, but are not limited to, “sentience”, “consciousness”, “intelligence”, “computation”, “cognition”, “qualia”, “agency” and “behavior”.

Secondly, identifying reliable objective metrics which can track phenomena of ethical relevance should remain a focus of research going forward(Goddard et al., 2023; Pereira et al., 2023; Smirnova et al., 2023). These can accord with challenges which fall under both the applications of SBI and the moral status of SBI as described above. It will be necessary to identify which candidates are necessary to consider for moral status. Functional markers such as being goal-directed, autonomously responsive, or showing learning can be considered as part of this. However, it should be noted that performing a function alone is not sufficient to identify a system as “phenomenologically conscious”. Examples of function without reported conscious experience have regularly been observed in Type 1 blindsight patients, who can perform relatively complex behaviors with no perception of the relevant sensation (Brogaard, 2011; Burra et al., 2019; Kagan et al., 2022a). As such, determining the moral status of SBI will require development of metrics that can help researchers infer when a model might develop these properties. Further, deciding the moral relevance of this status for a given application will also require agreement on what properties give rise to moral status and how best to proceed. Such an approach should involve a meaningful dialogue with the broader public and stakeholders to determine where ethical boundaries may lie.

Thirdly, once these understandings have been obtained, it will become crucial to identify areas and approaches that maximize benefits and minimize risks. Anticipatory approaches to the ethics and governance of emerging science and technologies could be useful here (Lysaght, 2022; Nielsen et al., 2015; Stahl, 2013). Anticipatory governance is one of several approaches to the ethics of emerging technologies, that aims to achieve socially and morally desirable outcomes from scientific research in the presence of high uncertainty(Kendal, 2022; Lysaght, 2022; Stahl, 2013). Anticipatory governance is most appropriate for technologies that are still in the process of emerging, where there is an absence of empirical models that can reliably predict risk and benefit (epistemic uncertainty) and where specialists in the field may not yet even agree on what the technology is (ontological uncertainty) to inform traditional ethical analyses. We favor this approach because, rather than reacting to uncertainty with overly precautious and prohibitive measures, anticipatory frameworks seek to explore and respond to emerging ethical and moral implications as the technology evolves from within the wider societal contexts they are situated.

For example, when considering what metrics may identify features worthy of moral consideration, it is likely that only through further development of SBI technology will the necessary knowledge to even identify these metrics be obtained. As described above, while currently some measures used in humans such as the PCI may have some merit, there is no evidence they are appropriate for *in vitro* systems. This inherent uncertainty further highlights the need for an approach that can reasonably anticipate morally significant properties and guide an ethical response as new evidence and knowledge emerges over obstructive precautionary measures that pay insufficient attention to the potential for beneficial outcomes. To manage the uncertainties and avoid polarizing the discussions, anticipatory approaches apply deliberative methods for engaging stakeholders and wider publics, as recently demonstrated with organoid research (Boyd and Sugarman, 2022)and human genome editing (Nelson et al., 2021).

## Conclusion

Elucidating the full range of applications and associated ethical or moral issues raised by SBIs exceeds the scope of this work. Therefore, here we have proposed key steps to building a viable framework to explore these issues in a constructive manner. Researchers should engage with broader publics and stakeholders to generate meaningful dialogue on the moral boundaries and shape SBI applications towards achieving socially and ethically desired outcomes.

Going forward, one key question will be: What, if anything, can we deduce about the moral status of these entities? For example, it has been argued that the most important feature of conscious systems that gives rise to moral status is not general, or domain specific, intelligence, but rather evaluative sophistication – the capacity to have a wide range of valanced subjective experiences(Shepherd, 2018). This builds on a view first articulated by Jeremy Bentham regarding the moral status of animals: “The question is not, Can they reason? nor, Can they talk? but, Can they suffer?"^142^ Following this perspective, even if SBIs produce human-like intelligence, this does not inherently imply they have moral status. Despite this, it is possible that the more sophisticated neural architecture required for human-like intelligence may facilitate more complex – and more morally valuable – conscious experiences and/or cognitive mental states. Determining measures that can help researchers infer when systems are likely to possess evaluative sophistication should be a goal of on-going research.

## Footnotes

1 Here “goal-directed or otherwise intelligent behavior” is used in a broad sense to refer to patterns of activity elicited in the biological substrate in response to, or as a result of, external stimulation that achieves an otherwise arbitrary effect in the external environment (simulated or otherwise). In other words, does the activity of the biological substrate lead to an outcome that would otherwise not have occurred but for the environmental input?

2 Here, embodied is taken to mean separated from the external environment. Here, embodiment means able to have an internal system to act and be acted upon via this external environment and which is enabled through a closed-loop system of information input and output. It does not denote any inherent capacity in and of itself.

3 This standardization effort should involve a public invitation to the broad scientific community to ensure a multi-disciplinary approach is adopted and to encourage widespread adoption. The authors have recently begun work into this endeavor and welcome collaborators to join.
